# Exosomal miR-141-3p from PDLSCs Alleviates High Glucose-Induced Senescence of PDLSCs by Activating the KEAP1-NRF2 Signaling Pathway

**DOI:** 10.1155/2023/7136819

**Published:** 2023-05-26

**Authors:** Min Liu, Rui Chen, Yunxuan Xu, Jiawen Zheng, Min Wang, Ping Wang

**Affiliations:** ^1^Department of Stomatology, The First Affiliated Hospital of Chongqing Medical University, Chongqing 400016, China; ^2^Department of Oral and Maxillofacial Surgery, The First Affiliated Hospital of Chongqing Medical University, Chongqing 400016, China

## Abstract

Human periodontal ligament stem cells (PDLSCs) are the most promising stem cells for periodontal tissue engineering. Senescent PDLSCs have diminished abilities to proliferate and differentiate, affecting the efficiency of periodontal tissue repair and regeneration. Stem cell-derived exosomes are important participants in intercellular information exchange and can help ameliorate senescence. In this study, we investigated PDLSC senescence in a high glucose microenvironment as well as the ability of human periodontal ligament stem cell-derived exosomes (PDLSC-Exos) to alleviate cellular senescence and the underlying mechanisms. Herein, PDLSCs and PDLSC-Exos were isolated and extracted. Then, cellular senescence indicators were evaluated after high glucose (25 mM) treatment of cultured PDLSCs. PDLSC-Exos were cocultured with senescent PDLSCs to further explore the role of PDLSC-Exos in cellular senescence and determine the differences in cellular oxidative stress levels after PDLSC-Exo treatment. Next, we investigated whether PDLSC-Exos alleviated cellular senescence by restoring the balance of oxidative stress signals and explored the underlying molecular pathways. We discovered that PDLSCs underwent premature senescence due to high glucose culture, but they were rejuvenated by PDLSC-Exos. The rejuvenating effects of PDLSC-Exos were notably reversed by cotreatment with ML385, an inhibitor of nuclear factor erythroid 2-related factor 2 (NRF2), indicating that this recovery depended on NRF2 activation. Further analyses revealed that microRNA-141-3p (miR-141-3p) was expressed at relatively high levels in PDLSC-Exos and was instrumental in PDLSC-Exo-mediated restoration by downregulating Kelch-like ECH-associated protein 1 (KEAP1), which is a negative regulator of NRF2 expression. Our findings suggest that PDLSC-Exos alleviate high glucose-induced senescence of PDLSCs by transferring miR-141-3p to activate the KEAP1-NRF2 signaling pathway. Based on this research, PDLSC-Exos may behave similarly to their parental PDLSCs and have significant effects on cellular senescence by delivering their encapsulated bioactive chemicals to target cells.

## 1. Introduction

In recent decades, diabetes and its complications have reached epidemic levels worldwide [1]. Periodontitis is the sixth most common complication of diabetes and the main cause of adult tooth loss, which seriously influences the health and quality of life of patients [2]. Treatment for diabetic periodontitis has achieved limited success in the clinic, but developments in tissue engineering and stem cell research have provided new opportunities for the restoration of periodontal tissue injury. Human periodontal ligament stem cells (PDLSCs), also known as undifferentiated mesenchymal stem cells (MSCs), are widely accepted as ideal seed cells for periodontal tissue regeneration [3]. According to previous reports, various factors influence the biological activity of PDLSCs, and hyperglycemia is one of the most common risk factors [4].

Cellular senescence is a permanently arrested state of the cell cycle evoked by either internal or external factors [5]. Hyperglycemia is a key extracellular stress signal that triggers cellular senescence. Hyperglycemia causes excessive oxidative stress, telomere shortening, epigenetic alterations, and mitochondrial dysfunction, thereby triggering the aging of MSCs [6, 7]. Multiple factors contribute to PDLSC senescence, such as transforming growth factor-*β* induction [8], long-term in vitro culture [9], and insulin-like growth factor binding protein 5 overexpression [10]. However, reports on the effect of high glucose levels on the senescence of PDLSCs are lacking. PDLSC senescence is accompanied by a reduced proliferative potential, impaired multipotent differentiation capabilities, and increased apoptosis [11, 12] and is not conducive to the repair and regeneration of periodontal defects. Accordingly, delaying cellular senescence may be an emerging option to activate intrinsic periodontal tissue repair mechanisms.

Exosomes are membrane-derived lipid bilayer-enclosed vesicles secreted by various cells that contain a variety of cell-derived lipids, proteins, and nucleic acids [13]. Exosomes can transport various signaling molecular components and modulate recipient cells through intercellular communication [14]. Stem cell-derived exosomes function similarly to the stem cells from which they originate and may represent a cellular replacement for tissue regeneration and ameliorate cellular senescence via multiple pathways [15, 16]. For instance, antler stem cell-derived exosomes could relieve osteoarthritis by counteracting the senescence of intra-articular cells [17]. Additionally, by transferring microRNA-200a (miR-200a) to stimulate the Kelch-like ECH-associated protein 1- (KEAP1-) nuclear factor erythroid 2-related factor 2 (NRF2) signaling pathway, exosomes produced from human embryonic stem cells were shown to alleviate the senescence of endothelial cells [18]. Human periodontal ligament stem cell-derived exosomes (PDLSC-Exos), which are the main components of PDLSC paracrine factors, have a wide range of activities, including healing periodontitis-related bone abnormalities and stimulating proliferation, angiogenesis, and immunomodulation [19–21]. However, the functions and underlying mechanisms of PDLSC-Exos in cellular aging are still unclear.

Thus, this study is aimed at investigating PDLSC senescence in a high glucose microenvironment as well as the effect and underlying mechanisms of PDLSC-Exos on alleviating cellular senescence. According to the oxidative stress theory of aging, enhancing the body's antioxidant defenses may be a therapeutic option to delay aging and extend lifespan [22]. One of the most important cellular antioxidant systems, the KEAP1-NRF2 pathway, controls the expression of many antioxidant enzymes [23]. According to previous studies, NRF2 signaling decreases in the aging process and the activation of NRF2 signaling can treat diseases associated with aging or stop cellular senescence, as well as slow the aging process [24, 25]. NRF2 activation prevents cellular senescence in MSCs [26], fibroblasts [27], and dendritic cells [28], reduces the burden of cardiovascular diseases in elderly individuals [29], and reverses the progression of age-associated fibrotic disorders [30]. Consequently, NRF2 activation may be important in ameliorating aging and related diseases. Mesenchymal stem cell-derived exosomes (MSC-Exos) have been reported to possess excellent antioxidant properties [31], such as protecting against oxidative stress-induced skin injury by regulating the NRF2 antioxidant defense system [32]. PDLSCs are dental stem cells that exhibit properties similar to those of MSCs. Thus, we surmise that PDLSC-Exos might have properties comparable to those of MSC-Exos and could inhibit cellular senescence under high-glucose conditions by alleviating oxidative stress through the NRF2 signaling pathway.

To test this hypothesis, we separated PDLSCs from the periodontal ligament and evaluated the effects of a high-glucose microenvironment on the senescence of PDLSCs. The ability of PDLSC-Exos to reduce oxidative stress and rescue cells from senescence was also assessed. Finally, we examined and validated the molecular mechanism regulating PDLSC senescence.

## 2. Materials and Methods

### 2.1. Cell Culture and Identification

PDLSCs were derived from healthy wisdom teeth or orthodontic teeth from young patients after obtaining informed consent from all patients. Phosphate-buffered saline (PBS) was used to clean the freshly extracted teeth, and the middle part of the root surface was scraped to collect the periodontal ligament tissue. Next, the tissues were digested with a type I collagenase solution (3 mg/mL) (Sigma-Aldrich, St. Louis, MO, USA) at 37°C for 15 min, followed by the addition of 5 mL of *α*-minimum essential medium with a penicillin–streptomycin solution (1%) and fetal bovine serum (FBS; Gibco, Grand Island, NY, USA) (10%). Afterward, tissues in the flask were cultured in a humid incubator with 5% CO_2_ at 37°C. The medium was replaced when the cells migrated out of the tissue, and third-generation cells were used for subsequent identification. With alizarin red staining, oil red O staining, and colony formation assays, the multidirectional differentiation potential and colony formation capacity of PDLSCs were validated.

#### 2.1.1. Alizarin Red Staining

Cells were seeded in 6-well plates (5 × 10^5^ cells/well). An osteogenic induction medium was added, and the medium was replaced every 2 days after the cells achieved 80% confluence. Twenty-one days following osteogenic induction, alizarin red staining (Beyotime Institute of Biotechnology, Shanghai, China) was performed. Cetylpyridinium chloride was used to liberate the calcified nodules, and the absorbance was measured at 562 nm.

#### 2.1.2. Oil Red O Staining

Cells were seeded in 6-well plates (5 × 10^5^ cells/well). The medium was replaced with an adipoinductive medium once the density reached 80%. The lipid droplets were stained with oil red O after 14 days. The stained lipid droplets were dissolved in isopropanol, and the absorbance was measured at 510 nm. Solarbio Co., Ltd. (Beijing, China) produced the kit utilized for this experiment.

#### 2.1.3. Colony Formation Assay

Cells were seeded in 60 mm culture dishes (800 cells/plate). Following 14 days of continuous culture, 0.1% crystal violet was added to stain the cells that had been fixed with 4% paraformaldehyde (PFA). The excess dye was rinsed off, and the cells were observed under a microscope. Cell aggregates containing more than 50 cells were considered colonies.

### 2.2. Effect of High Glucose Levels on PDLSC Senescence

Second passage PDLSCs from the same donor were continuously cultured in a high-glucose medium (HG group, 25 mM glucose) to explore the effect of a high-glucose environment on PDLSC senescence. Every two days, the medium was changed, and the cells were passaged when they reached 80% confluence. The control cells were cultured in high-mannitol (HM group, an osmotic control, 5.5 mM glucose + 19.5 mM mannitol) or normal glucose medium (NG group, 5.5 mM glucose). The cells in each group were monitored for senescence-associated *β*-galactosidase (SA-*β*-gal) activity at various intervals (7, 14, 21, and 28 days) to ascertain the ideal time of high-glucose treatment to induce cellular senescence. Apoptotic morphology in the HG group was observed by performing Hoechst 33258 staining at different time points (7, 14, 21, and 28 days) to differentiate apoptosis from cellular senescence. In subsequent investigations, cells from each group that had been continuously cultured for 14 days were employed. The expression of senescence-associated proteins (p53, p21, and p16) was evaluated using western blotting (WB) or immunofluorescence (IF) staining to further assess cellular senescence. Quantitative real-time PCR (qRT–PCR) analysis was performed to assess the senescence-associated secretory phenotype (SASP) components interleukin 6 (*IL-6*) and interleukin 8 (*IL-8*).

We next measured cell proliferation, migration, and multidirectional differentiation to assess the changes in cellular biological functions after the induction of senescence by high glucose levels. Cell proliferation was assessed using Cell Counting Kit-8 (CCK-8) and clone formation assays (see the section “Colony formation assay”); wound healing and transwell assays were performed to detect cell migration; and alkaline phosphatase (ALP), alizarin red staining (see the section “Alizarin red staining”), oil red O (see the section “Oil red O staining”) staining, and quantitative detection of cellular multidirectional differentiation potential were performed.

#### 2.2.1. SA-*β*-Gal Staining

An SA-*β*-gal staining kit was used to identify SA-*β*-gal activity in certain groups of fixed cells. Microscopic observations showed that SA-*β*-gal-positive cells were blue. From each sample, three fields (each with at least 100 cells) were chosen at random to calculate the percentage of SA-*β*-gal positive cells as follows: (*β* − gal − positive cells/total cells in a field) × 100%. The kit utilized in this investigation was created by Beyotime Institute of Biotechnology Co., Ltd. (Shanghai, China).

#### 2.2.2. Hoechst 33258 Staining

An apoptosis-Hoechst staining kit was applied to observe the apoptosis of PDLSCs induced by high glucose levels. At appropriate time intervals, the cells were fixed and stained with Hoechst 33258 staining solution. The nuclei of the cells were observed under a fluorescence microscope. Beyotime Institute of Biotechnology Co., Ltd. (Shanghai, China) produced the kit utilized in this experiment.

#### 2.2.3. Immunofluorescence Staining

Each batch of cells was grown in 24-well plates and preserved with 4% PFA when the cell density reached approximately 70%. Goat serum (Beyotime Institute of Biotechnology, Shanghai, China) was utilized to block the cells for 30 min after they had been permeabilized with 0.1% Triton X-100 for 10 min. Cells were incubated with primary antibodies overnight at 4°C followed by secondary antibodies for one hour at room temperature. Then, the nuclei were stained with 4′,6-diamidino-2-phenylindole (DAPI). A fluorescence microscope was employed to observe the cells. The antibodies utilized in this experiment are listed in Table S1.

#### 2.2.4. Western Blotting

RIPA strong buffer (containing protease and phosphatase inhibitors) and a nuclear and cytoplasmic protein extraction kit (Beyotime Institute of Biotechnology, Shanghai, China) were used to extract total protein and nuclear protein, respectively. A bicinchoninic acid (BCA) assay kit was employed to calculate the protein concentration. Electrophoresis on polyacrylamide gels with sodium dodecyl sulfate was employed to separate protein samples. Proteins were transferred to PVDF membranes, which were then blocked for two hours at room temperature with 5% skim milk. The primary antibody was applied to detect the protein bands and incubated overnight at 4°C, followed by an hour of incubation with the secondary antibody at room temperature. Finally, proteins were detected using an enhanced chemiluminescence reagent (BioSharp, Hefei, China). Spectral density analysis was performed using ImageJ software. In this experiment, the internal reference for total cellular protein was GAPDH, whereas histone H3 served as the internal standard for quantifying nuclear protein. Goat anti-rabbit IgG horseradish peroxidase (1 : 5000, Affinity Biosciences, Jiangsu, China) was utilized as the secondary antibody. The antibodies that were employed in this experiment are displayed in Table S1.

#### 2.2.5. qRT–PCR Analysis of mRNA Levels

TRIzol was applied to extract total cellular RNA, and a reverse transcription kit was employed to synthesize cDNAs. With SYBR Premix Ex Taq™ II, a quantitative real-time polymerase chain reaction was performed. The expression of major SASP factors, including *IL-6* and *IL-8*, was detected. The internal control was GAPDH. TaKaRa (Tokyo, Japan) produced the kits utilized in this investigation, and sequences of the primers used to detect each gene sequence are listed in Table S2.

#### 2.2.6. CCK-8 Assay

The CCK-8 assay was performed using a CCK-8 kit (ApexBio Technology, MA, USA). Cells were seeded in 96-well plates (1 × 10^3^ cells/well), and each well received 10 *μ*L of CCK-8 test solution and 100 *μ*L of the medium before incubation at 37°C for two hours. The absorbance values (OD values) were measured at 450 nm with a microplate reader.

#### 2.2.7. Wound Healing Assay

Cells were seeded in 6-well plates. Wounds were created by scraping the cell monolayers with 200 *μ*L pipette tips. An optical microscope captured photos at predetermined time intervals (0 h, 24 h, and 48 h) after scratching. Calculations were performed based on the initial scratch area to determine how the scratch area changed over time and what proportion of the wound had healed. Wound healing rate (%) = (scratch area at 0 h − scratch area at a particular time point)/scratch area at 0 h × 100.

#### 2.2.8. Transwell Migration Assay

Each group of cells (4 × 10^4^ cells/well) was plated in the top chambers of transwell membranes in a 24-well plate, and media containing 10% exosome-free FBS (750 *μ*L/well) was added to the lower chambers. Cells were preserved with 4% PFA after a 24 h incubation and stained with 0.1% crystal violet. Afterward, the cells on the membrane surface were removed, and an inverted microscope was utilized to capture images of the dyed cells. The cells stained with crystal violet were washed with 33% acetic acid, and the absorbance of the solution was measured at OD 570 nm.

#### 2.2.9. ALP Staining and Activity Assay

Cells were plated in 6-well culture dishes at a density of 5 × 10^5^ cells. After the cells reached 80% confluence, the medium was replaced with an osteoinductive medium. ALP staining was performed after one week of osteoinductive culture. An ALP assay kit from the Beyotime Institute of Biotechnology Co., Ltd. (Shanghai, China) was employed for the quantitative measurement of ALP levels.

### 2.3. Exosome Isolation and Characterization

Exosomes were extracted using differential centrifugation/ultracentrifugation protocols. Young PDLSCs were cultured in a complete medium. When the cell density reached 70%, the medium was replaced with a serum-free medium, cells were cultured for 48 h, and the cell supernatant was collected. After the conditioned medium was centrifuged at 300 × g for 15 min, the supernatant was removed, and the samples were centrifuged again for 15 min at 3,000 × g. The resulting supernatant was centrifuged twice at 100,000 × g for 70 min each. After the supernatant was removed, the exosome-containing particles were resuspended in cold PBS and stored at -80°C until use in subsequent experiments. In experiments involving exosomes, the same volume of PBS was utilized as a negative control.

The concentration of exosomes was determined with a BCA assay kit. The morphology of PDLSC-Exos was characterized using transmission electron microscopy (TEM). The particle size and concentration of the exosomes were assessed using nanoparticle tracking analysis (NTA). WB was used to identify exosome surface markers such as CD9, CD63, CD81, TSG101, and calnexin (see the section “Western blotting”).

### 2.4. Effects of PDLSC-Exo Treatment on Senescent PDLSCs

We first determined whether PDLSC-Exos were taken up by senescent PDLSCs. Senescent PDLSCs were treated with 25, 50, and 100 *μ*g/mL PDLSC-Exos for 72 h to identify a suitable PDLSC-Exo concentration. The response of high glucose-induced cellular senescence to PDLSC-Exo treatment was assessed by performing SA-*β*-gal staining (see the section “SA-*β*-gal staining”). Afterward, we selected 50 *μ*g/mL PDLSC-Exos and coincubated them with senescent PDLSCs for 72 h (HG-Exos group), while PDLSCs in the control groups received a similar volume of PBS (HG group) or normal glucose (NG group, without high-glucose treatment). Similarly, senescence-associated protein expression was detected using WB (see the section “Western blotting”) or IF staining (see the section “Immunofluorescence staining”). Additionally, qRT–PCR was implemented to assess the expression of the indicated proinflammatory SASP genes (see the section “qRT–PCR analysis of mRNA levels”). We examined cell proliferation, migration, and multidirectional differentiation to determine the changes in cellular biological functions (see the section “Effect of high glucose levels on PDLSC senescence”).

#### 2.4.1. Labeling of Exosomes and Uptake by PDLSCs

Exosomes were labeled with the fluorescent dye Dil (red) to evaluate PDLSC-Exo uptake by senescent PDLSCs. Briefly, 2 *μ*L of Dil was added to the isolated exosomes (1 mL, 100 *μ*g/mL protein concentration) to a final concentration of 10 *μ*m. Dil-labeled exosomes were reisolated after a 30 min incubation at 37°C. Senescent PDLSCs were incubated with the labeled exosomes for 24 h at 37°C. Next, the cells were fixed, the nuclei were stained with DAPI, and an inverted fluorescence microscope was used to capture images of the ingested exosomes. The reagent used in this study was created by the Beyotime Institute of Biotechnology Co., Ltd. (Shanghai, China).

### 2.5. Role of NRF2 in the PDLSC-Exo-Mediated Rejuvenation of Senescent PDLSCs

Before and after PDLSC-Exo treatment of senescent PDLSCs, we measured the reactive oxygen species (ROS) level, malondialdehyde (MDA) content, and superoxide dismutase (SOD) activity to evaluate cellular oxidative stress. We employed WB to identify the expression of the NRF2 and NRF2-downstream target proteins NADPH quinone oxidoreductase 1 (NQO1) and heme oxygenase 1 (HO-1) in cells to examine the involvement of NRF2 in PDLSC-Exo-mediated rejuvenation of senescent PDLSCs (see the section “Western blotting”). In addition, the expression and intracellular distribution of NRF2 were determined by IF staining (see the section “Immunofluorescence staining”).

High glucose-induced senescent PDLSCs were cultured in the following groups to explore the effects of NRF2 activation on the PDLSC-Exo-mediated restoration of senescent PDLSCs: (1) HG group (PBS treatment), (2) HG-Exos group (50 *μ*g/mL PDLSC-Exo treatment), and (3) HG-Exos-ML385 (MedChem Express, Monmouth Junction, NJ, USA) group (50 *μ*g/mL PDLSC-Exos and 5 *μ*m ML385). Similarly, NRF2 levels and localization in each group were detected as described above. Then, cellular senescence, oxidative stress, and biological functions were successfully examined to evaluate the effectiveness of ML385 treatment in restoring PDLSC-Exo-mediated cell viability.

#### 2.5.1. Measurement of Oxidative Stress Levels

An ROS assay kit was employed to measure intracellular ROS levels according to the manufacturer's instructions. Briefly, the cells received 10 *μ*mol/L DCFH-DA and were incubated at 37°C for 20 min. Fluorescence microscopy and a microplate reader were utilized to analyze the fluorescence intensity and assess the formation of ROS. For the determination of MDA levels and SOD activity, cell supernatants were first collected after cell lysates were generated. With a commercial kit, the MDA level and SOD activity were determined according to the manufacturer's procedure. Beyotime Institute of Biotechnology Co., Ltd. (Shanghai, China) produced the kits utilized in this experiment.

### 2.6. Role of the microRNA-141-3p (miR-141-3p)/KEAP1/NRF2 Axis in PDLSC-Exo-Mediated Cellular Rejuvenation

KEAP1 (an NRF2 negative regulatory protein) expression levels in senescent PDLSCs and changes in its expression following PDLSC-Exo therapy were determined using WB (see the section “Western blotting”). Then, we examined miRNAs that specifically target KEAP1 to regulate NRF2 activity. Using qRT–PCR, we measured the expression levels of two screened miRNAs (miR-141-3p and miR-200a-3p) in young and senescent PDLSCs and PDLSC-Exos. The expression of miR-141-3p was then evaluated following the PDLSC-Exo treatment of senescent PDLSCs. The interaction between KEAP1 and miR-141-3p was verified by performing a dual-luciferase reporter experiment. We downregulated the expression of miR-141-3p in PDLSC-Exos using a miR-141-3p inhibitor to validate the molecular mechanism by which PDLSC-Exos regulate the NRF2 signaling pathway. Then, high glucose-induced senescent PDLSCs were divided into three groups: (1) the HG group (PBS treatment), (2) the HG-NCI-Exos group (50 *μ*g/mL NCI-Exo treatment, exosomes isolated from PDLSCs transfected with inhibitor negative control), and (3) the HG-141I-Exos group (50 *μ*g/mL 141I-Exo treatment, exosomes isolated from PDLSCs transfected with the miR-141-3p inhibitor). According to the aforementioned classification, follow-up experiments were conducted. WB (see the section “Western blotting”) and IF staining (see the section “Immunofluorescence staining”) were performed to identify the expression of proteins in the NRF2 pathway. Additionally, we evaluated the senescence, oxidative stress, and biological functions of the cells from each group.

#### 2.6.1. miRNA Screening

With a Venn plot, candidate miRNAs targeting *KEAP1* were screened based on cross-filtering of miRNA databases (miRWalk [33], StarBase [34], miRDB [35], miRcode [36], and TargetScan [37]). Moreover, the keywords “microRNA, cellular senescence, periodontal ligament stem cells, periodontal disease, oxidative stress, mesenchymal stem cells, and KEAP1-NRF2” were searched in PubMed to identify whether these previously screened miRNAs were expressed in periodontal tissue and involved in cellular senescence, oxidative stress, and periodontal disease.

#### 2.6.2. qRT–PCR Analysis of miRNA Levels

The BIOG Exosome RNA Easy Kit was applied to extract total exosomal RNA, and the BIOG miRNA PolyA+ RT Kit was employed to reverse transcribe miRNAs. qRT–PCR was performed using the BIOG miRNA PolyA SYBR qPCR kit. The expression of the target genes, including miR-141-3p and miR-200a-3p, was measured using qRT–PCR. The internal control for the miRNA analysis was U6. The kits used in this experiment were created by Changzhou Baidai Biotechnology Co., Ltd. (China), and each primer sequence is listed in Table S3.

#### 2.6.3. Dual-Luciferase Reporter Assay

The National Center for Biotechnology Information database was searched to identify the 3′ untranslated region (UTR) of *KEAP1* mRNA, and the sequence was generated and cloned downstream of the luciferase minigene in the pmirGLO vector (luciferase reporter vector). With a site-directed mutagenesis kit (Umibio, Shanghai, China), the 3′UTR of the putative miR-141-3p target sequence was altered. Each product was sequenced. The miR-141-3p sequence was also determined and synthesized. HEK293 cells that did not express the target miR-141-3p were plated in 48-well plates (1 × 10^5^ cells/well) the day before transfection. As directed by the manufacturer, Lipofectamine 2000 (Invitrogen, Carlsbad, CA, USA) was employed for transfection. Cells were transfected with a pmirGLO luciferase expression construct containing the 3′UTR of the *KEAP1* gene, pRL-TK Renilla luciferase vector (Promega, Madison, WI, USA), and miRNA negative control (Ambion, Austin, TX, USA). With a dual-luciferase reporter assay kit from Promega (Madison, WI, USA), luciferase activities were assessed 48 h after transfection and normalized to Renilla luciferase activity.

#### 2.6.4. Transfection of the miRNA Inhibitor

According to the manufacturer's instructions, PDLSCs were transfected with 100 nM miR-141-3p inhibitor (Ribo Bio, Guangzhou, China) and inhibitor negative control using Lipofectamine RNAiMAX (Thermo Fisher Scientific, Waltham, MA, USA). Transfected cells were grown in serum-free media for 48 h. A previously described approach was used to isolate exosomes from the cell culture supernatant.

### 2.7. Statistical Analysis

PDLSCs from the same donor were utilized in each experiment, which was conducted separately at least three times. GraphPad Prism 9 software was employed for statistical analysis. All results are presented as the means ± standard deviations (SD). Student's *t*-test was applied to determine the statistical significance of differences between groups. Analysis of variance (ANOVA) was used for multiple comparisons. Each experiment was repeated with PDLSCs from a minimum of three separate donors. Differences were considered statistically significant at *p* < 0.05 (^∗^), *p* < 0.01 (^∗∗^), *p* < 0.001 (^∗∗∗^), or *p* < 0.0001 (^∗∗∗∗^).

## 3. Results

### 3.1. Characterization of PDLSCs and PDLSC-Exos

**Figure 1:**
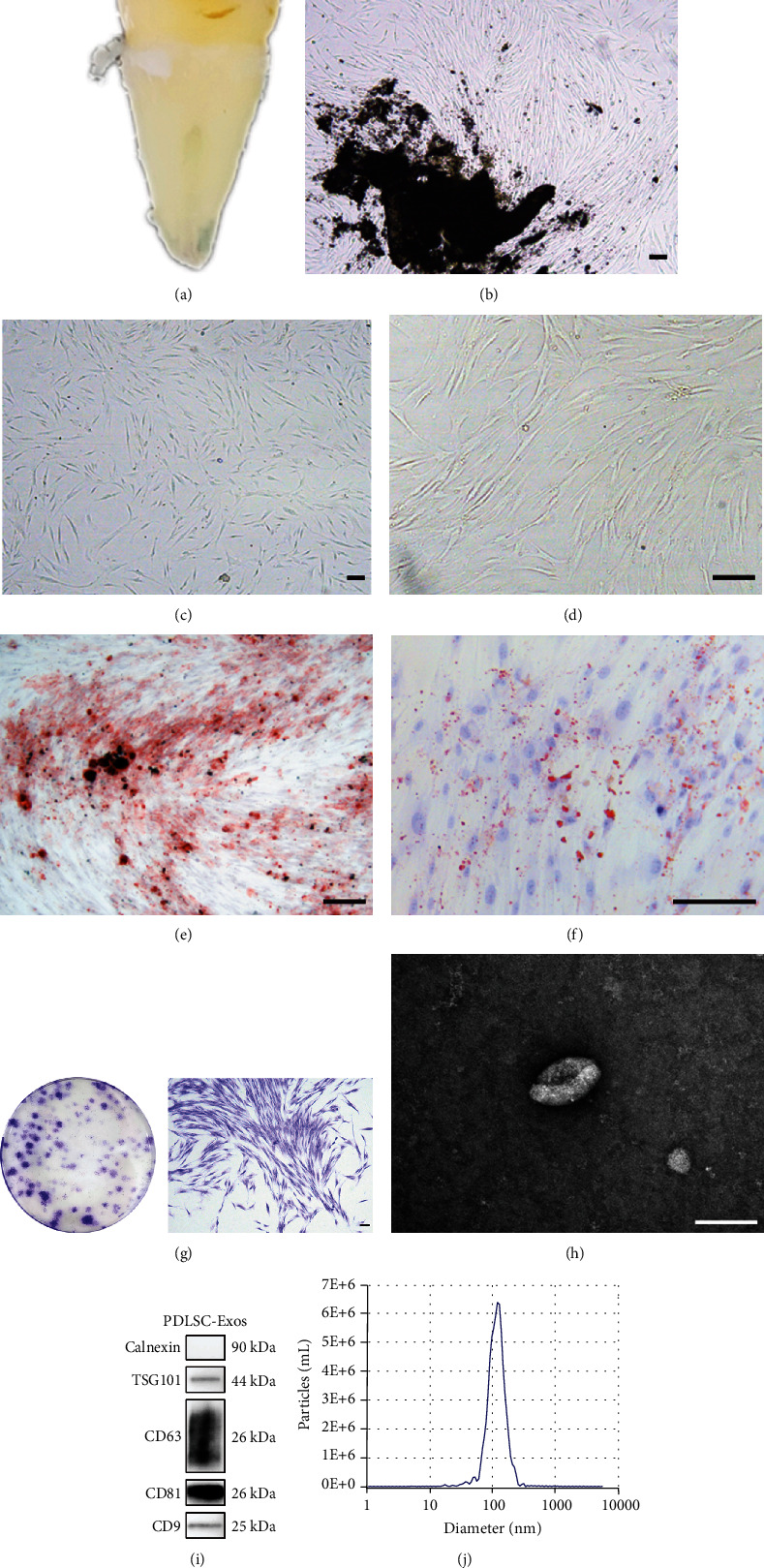
Characterization of human PDLSCs and PDLSC-Exos. (a–d) Isolation and passage of human PDLSCs. The cells exhibited a classic spindle-shaped morphology. Scale bar, 100 *μ*m. (e) Alizarin red staining of calcified nodules indicated the capacity of PDLSCs to develop into osteoblasts. Scale bar, 100 *μ*m. (f) Oil red O staining indicated the adipogenic differentiation capacity. Scale bar, 100 *μ*m. (g) Clone formation assay showing the proliferation of human PDLSCs. Scale bar, 100 *μ*m. (h) TEM observations revealed the typical cup-like shape of PDLSC-Exos. Scale bar, 100 nm. (i) WB showed that PDLSC-Exos expressed exosome marker proteins. (j) NTA revealed the particle size and concentration of PDLSC-Exos.

PDLSCs were derived from young healthy teeth (Figure 1(a)). Primary cells grew from the tissue center to the periphery (Figure 1(b)). After the subculture, the cells grew vigorously and displayed a typical fibroblast-like morphology with a whirlpool-like array (Figures 1(c) and 1(d)). Alizarin red and oil red O staining confirmed the osteogenic (Figure 1(e)) and adipogenic (Figure 1(f)) differentiation of PDLSCs. The stemness of the cells was confirmed by performing a plate clone formation assay (Figure 1(g)).

PDLSC-Exos were purified from the supernatants of young PDLSCs and further characterized using TEM, WB, and NTA. TEM analysis revealed that the PDLSC-Exos had a typical cup-like morphology (Figure 1(h)). WB showed that PDLSC-Exos expressed the exosome-specific markers CD9, CD81, CD63, and TSG101 but not calnexin (Figure 1(i)). According to the NTA data, the concentration of PDLSC-Exos was 3.9 × 10^10^ particles/mL with an average particle size of 113.8 nm (Figure 1(j)). These findings show that the PDLSC-Exos we extracted had the basic characteristics of exosomes.

### 3.2. High Glucose Induces Premature Senescence in PDLSCs

**Figure 2:**
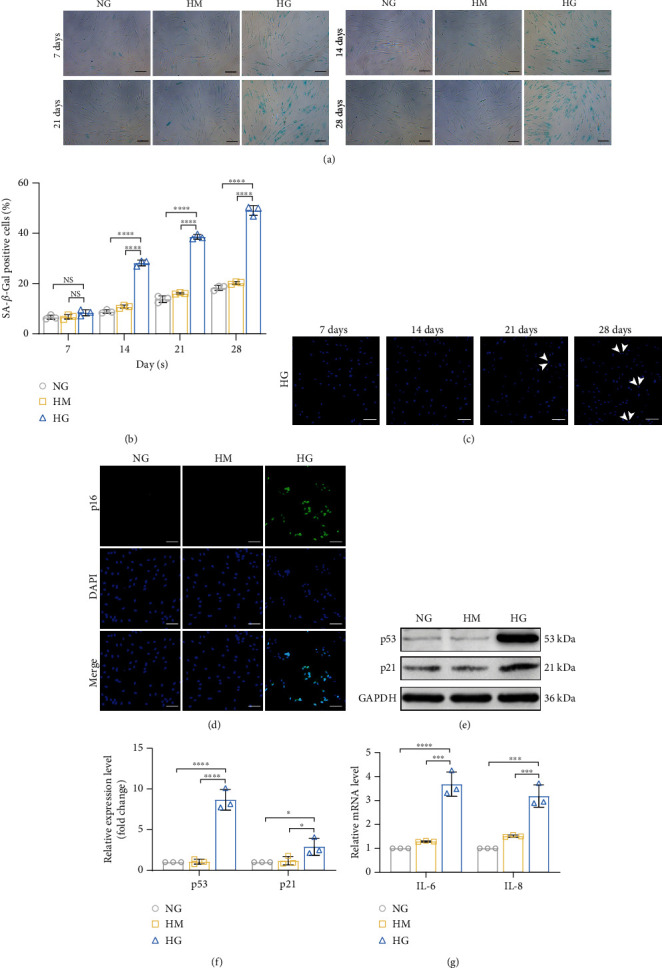
High glucose-induced senescence in human PDLSCs. PDLSCs were treated with normal glucose (NG group; 5.5 mM glucose), high mannitol (HM group; 5.5 mM glucose + 19.5 mM mannitol), and high glucose (HG group; 25 mM glucose) to observe phenotypic changes in senescence. (a) After 14 days of high glucose administration, an increase in the proportion of SA-*β*-gal-positive cells (blue) was clearly visible after SA-*β*-gal labeling. Scale bar, 100 *μ*m. (b) Quantification of the percentages of SA-*β*-gal-positive PDLSCs in the indicated groups. (c) Cell apoptosis was observed by Hoechst 33258 staining, and apoptotic nuclei were detected as condensed (white arrows). Scale bar, 100 *μ*m. (d) Expression of p16 (green), as measured using IF staining. Scale bar, 100 *μ*m. (e, f) WB and quantitative analyses of cellular senescence-related protein expression (p53 and p21). (g) The expression of the indicated SASP (*IL-6* and *IL-8*) genes was quantified using qRT–PCR. All data are presented as the means ± SDs (*n* = 3 independent observations). NS indicates not significant, ^∗^*p* < 0.05, ^∗∗∗^*p* < 0.001, and ^∗∗∗∗^*p* < 0.0001. The corresponding control is indicated.

After high-glucose treatment for 14 days, a significant increase in the number of SA-*β*-gal-positive cells was observed compared to those of the NG and HM groups (Figures 2(a) and 2(b)), but significant apoptosis was not observed (Figure 2(c)). Therefore, we chose 14 days of induction as the time point for the high-glucose treatment of PDLSCs in subsequent experiments. The levels of senescence-associated proteins (p53, p21, and p16) (Figures 2(d)–2(f)) and two major indicators of SASP (*IL-6* and *IL-8*) (Figure 2(g)) were also substantially increased following high-glucose induction. The results of the experiments assessing biological functions revealed decreased proliferation, migration, and osteogenic and adipogenic differentiation of high glucose-induced senescent PDLSCs (Supplementary Figure 1). Based on these results, continuous culture in a high-glucose medium induces premature senescence in PDLSCs.

### 3.3. PDLSC-Exos Alleviate High Glucose-Induced Senescence of PDLSCs

**Figure 3:**
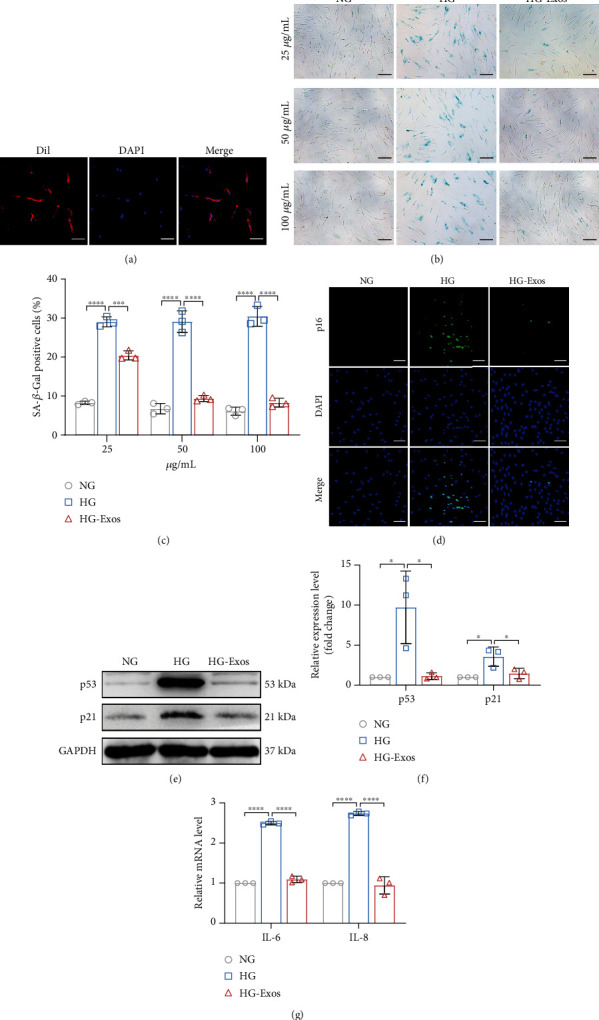
PDLSC-Exos ameliorate high glucose-induced senescence of human PDLSCs. PDLSCs were treated with high glucose levels to induce senescence and then treated with PDLSC-Exos (HG-Exos group) or PBS (HG group), while PDLSCs treated with normal glucose levels served as the control (NG group). (a) Efficient uptake of Dil-labeled exosomes by senescent PDLSCs was detected at 24 h. Scale bar, 100 *μ*m. (b) Images of SA-*β*-gal-stained cells. Scale bar, 100 *μ*m. (c) Quantification of the percentages of SA-*β*-gal-positive PDLSCs in the indicated groups. (d) IF detection of p16 expression (green). Scale bar, 100 *μ*m. (e, f) The expression of p53 and p21, two senescence-related proteins, was measured and evaluated using WB. (g) The expression of the indicated SASP genes (*IL-6* and *IL-8*) was determined using qRT–PCR. All data are presented as the means ± SDs (*n* = 3 independent observations). ^∗^*p* < 0.05, ^∗∗∗^*p* < 0.001, and ^∗∗∗∗^*p* < 0.0001. The corresponding control is indicated.

To further determine the effect of PDLSC-Exos on delaying cellular senescence, we first used Dil-labeled exosomes to test the capacity of PDLSC-Exo internalization. Red fluorescence was observed around the nuclei of PDLSCs cocultured with exosomes, indicating the presence of Dil-labeled PDLSC-Exos (Figure 3(a)); the results suggested that PDLSC-Exos entered senescent PDLSCs via endocytosis and were dispersed in the cytoplasm. According to SA-*β*-gal staining, PDLSC-Exo treatment substantially reduced SA-*β*-gal activity in senescent PDLSCs in a concentration-dependent manner (Figures 3(b) and 3(c)). In particular, the percentage of SA-*β*-gal-positive staining in the HG-Exos group was markedly reduced and similar to that in the NG group when the concentration of PDLSC-Exos reached 50 *μ*g/mL. Therefore, we used 50 *μ*g/mL PDLSC-Exos for subsequent experiments, and an equal volume of PBS was used as a placebo in the other groups. Moreover, PDLSC-Exos decreased the expression levels of p53, p21, and p16 (Figures 3(d)–3(f)), as well as the levels of *IL-6* and *IL-8* (Figure 3(g)), in senescent PDLSCs.

**Figure 4:**
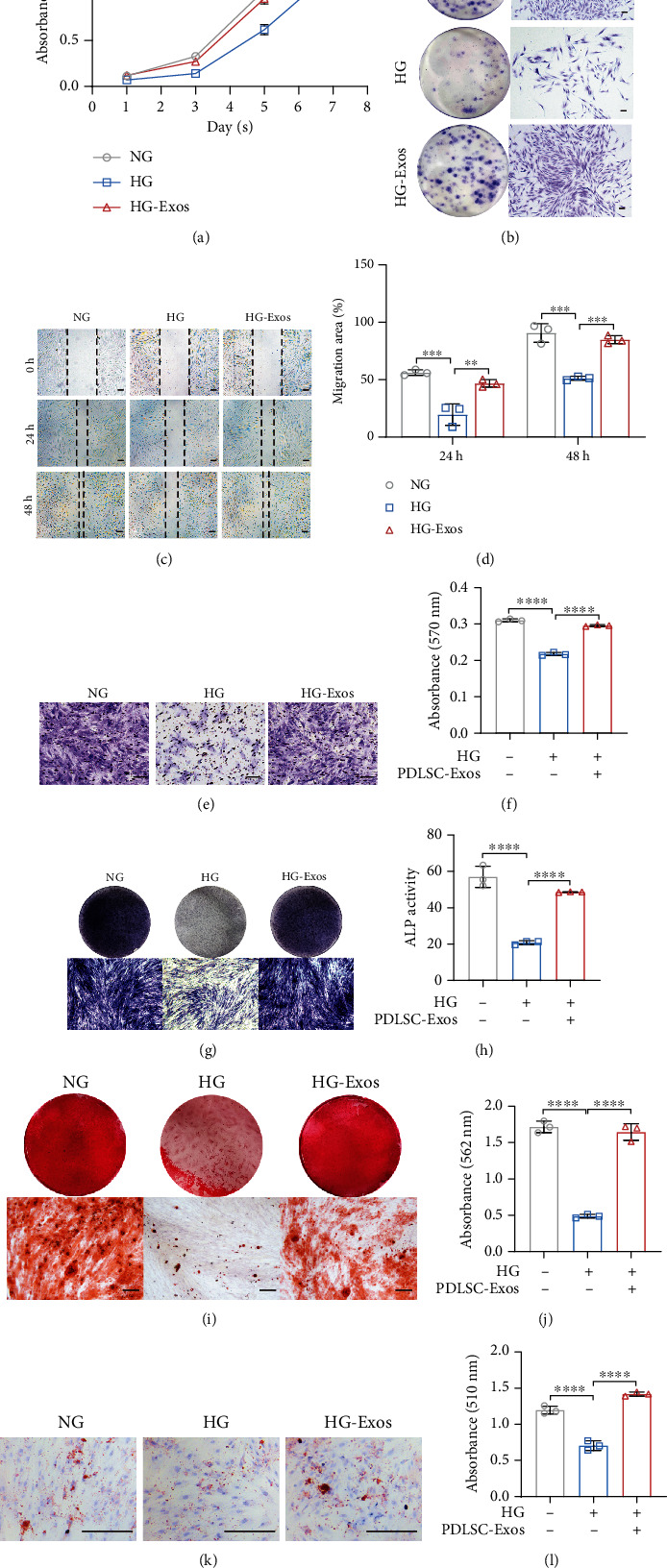
PDLSC-Exos attenuate the dysfunction of high glucose-induced senescent human PDLSCs. (a, b) CCK-8 and plate colony formation assays were utilized to detect the ability of PDLSCs to proliferate. Scale bar, 100 *μ*m. Through wound healing (c, d) and transwell experiments (e, f), cell migration was evaluated. Scale bar, 100 *μ*m. (g, h) ALP staining/activity assays. Scale bar, 100 *μ*m. (i, j) Alizarin red staining and quantification of mineralized nodules. Scale bar, 100 *μ*m. (k, l) Oil red O staining and quantification of the adipogenic differentiation capacity of the cells in the three groups. Scale bar, 100 *μ*m. All data are presented as the means ± SDs (*n* = 3 independent observations). ^∗∗^*p* < 0.01, ^∗∗∗^*p* < 0.001, and ^∗∗∗∗^*p* < 0.0001. The corresponding control is indicated.

Because the biological functions of PDLSCs deteriorate during the process of aging [12], we next examined the effects of PDLSC-Exo treatment on the biological properties of high glucose-induced senescent PDLSCs. The results of the CCK-8 assay (Figure 4(a)) and plate colony formation assay (Figure 4(b)) suggested that incubation with PDLSC-Exos restored the proliferation of high glucose-induced aged PDLSCs. Both the wound healing (Figures 4(c) and 4(d)) and transwell experiments (Figures 4(e) and 4(f)) indicated that PDLSC-Exo treatment may improve the decreased migratory potential of senescent PDLSCs. Moreover, the incubation of senescent PDLSCs with PDLSC-Exos improved the osteogenic (Figures 4(g)–4(j)) and adipogenic differentiation of the cells (Figures 4(k) and 4(l)). Overall, these findings showed that PDLSC-Exo therapy reduced the senescent phenotypes of PDLSCs and restored the vitality of old PDLSCs to a level that was approximately equivalent to that of young cells.

### 3.4. PDLSC-Exos Rejuvenate Senescent PDLSCs by Activating NRF2

**Figure 5:**
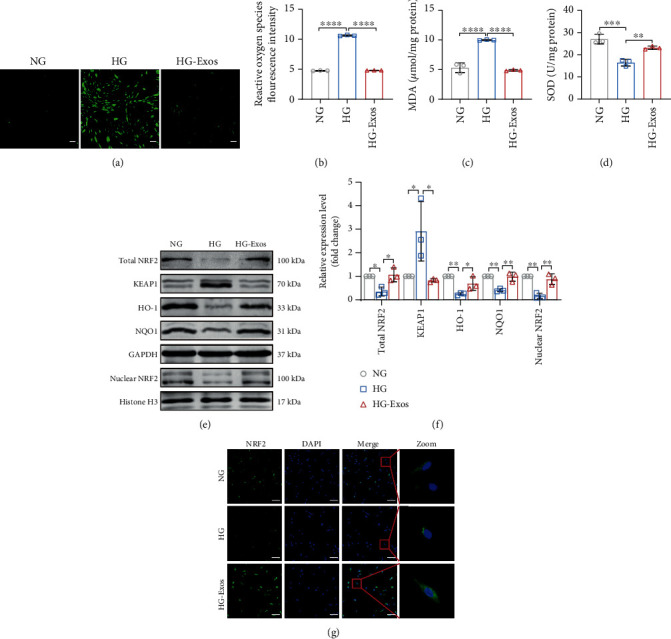
PDLSC-Exos decrease oxidative stress and increase the activity of the endogenous NRF2 antioxidant system. (a, b) Intracellular ROS levels were evaluated by measuring DCFH-DA fluorescence. Scale bar, 100 *μ*m. (c, d) The level of oxidative stress in cells was measured by detecting MDA levels and SOD activity. (e, f) Protein expression levels of total NRF2, nuclear NRF2, KEAP1, HO-1, and NQO1 in the indicated groups. (g) Localization of NRF2 (green), as visualized using IF staining. Scale bar, 100 *μ*m. All data are presented as the means ± SDs (*n* = 3 independent observations). ^∗^*p* < 0.05, ^∗∗^*p* < 0.01, ^∗∗∗^*p* < 0.001, and ^∗∗∗∗^*p* < 0.0001. The corresponding control is indicated.

The most common type of stress that causes cellular senescence in vitro is oxidative stress [38]. We first examined ROS levels in PDLSCs to determine the mechanism by which PDLSC-Exos exerted their antiaging effect. As shown in Figures 5(a) and 5(b), the intracellular ROS level in high glucose-induced senescent PDLSCs was substantially increased, but this effect almost completely disappeared after incubation with PDLSC-Exos. We also measured the MDA levels and SOD activity and found that the MDA level in the HG group was substantially greater than that in the NG group (Figure 5(c)), whereas the antioxidant activity of SOD was markedly lower (Figure 5(d)). After treatment with PDLSC-Exos, these changes in aged PDLSCs were largely reversed. Thus, PDLSC-Exos reduce oxidative stress in senescent PDLSCs. NRF2 plays a critical role in regulating redox and metabolic homeostasis, oxidative stress, and other cytoprotective responses [39]. We subsequently investigated the antiaging mechanisms of PDLSC-Exos on senescent PDLSCs by assessing whether PDLSC-Exos might promote NRF2 nuclear translocation and activate its downstream signaling pathway. According to the findings of our investigation, aged PDLSCs exhibited lower expression levels of NRF2 signaling-related proteins (NRF2, NQO1, and HO-1). PDLSC-Exo treatment restored the expression levels of NRF2 signaling-related proteins (Figures 5(e) and 5(f)) and promoted NRF2 nuclear translocation (Figure 5(g)) in senescent cells.

**Figure 6:**
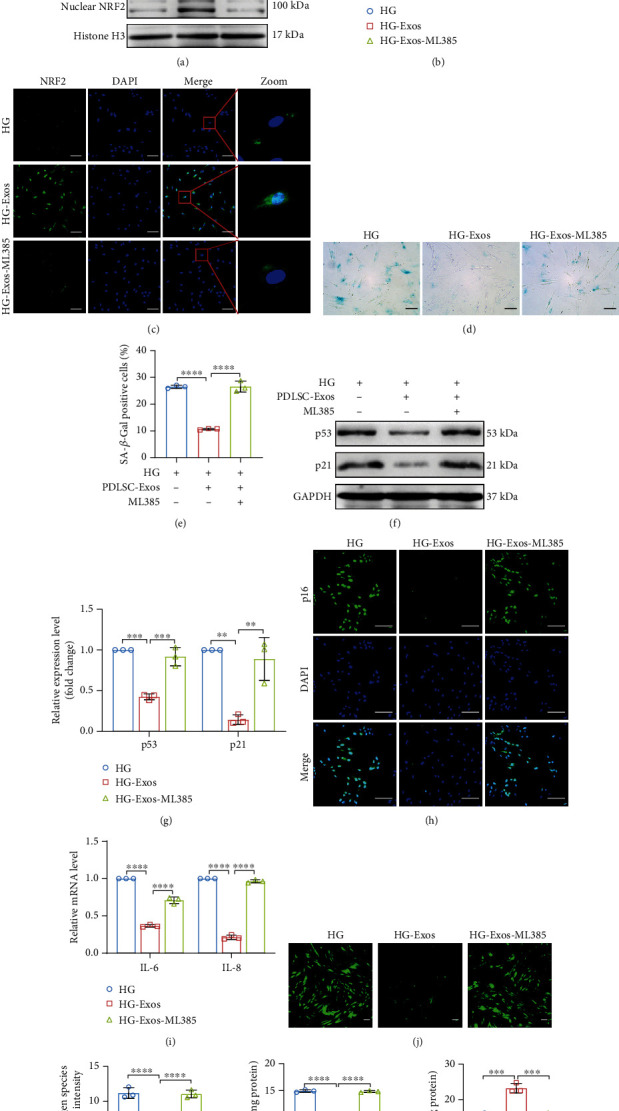
PDLSC-Exos rejuvenate senescent human PDLSCs by activating NRF2. High glucose-induced senescent human PDLSCs were treated with either PDLSC-Exos alone (HG-Exos group) or in combination with an NRF2 inhibitor (HG-Exos-ML385 group), while untreated senescent PDLSCs were used as controls (HG group). (a, b) WB analysis of total NRF2, nuclear NRF2, HO-1, and NQO1 levels in the indicated groups. (c) IF staining to detect the expression and localization of NRF2 (green). Scale bar, 100 *μ*m. (d, e) Cells were stained with SA-*β*-gal, and activity was determined by counting the number of SA-*β*-gal-positive cells. Scale bar, 100 *μ*m. (f, g) WB and quantification of senescence-related proteins (p53 and p21). (h) IF staining indicating p16 expression. Scale bar, 100 *μ*m. (i) SASP gene (*IL-6* and *IL-8*) expression levels were assessed using qRT–PCR. (j, k) Cellular ROS levels were detected and quantified by performing DCFH-DA staining. Scale bar, 100 *μ*m. (l, m) MDA levels and SOD activity were determined. All data are presented as the means ± SDs (*n* = 3 independent observations). ^∗^*p* < 0.05, ^∗∗^*p* < 0.01, ^∗∗∗^*p* < 0.001, and ^∗∗∗∗^*p* < 0.0001. The corresponding control is indicated.

Aged PDLSCs were incubated with the NRF2-specific inhibitor ML385 and PDLSC-Exos to further determine whether PDLSC-Exos delayed aging by upregulating NRF2. The results confirmed that ML385 prevented PDLSC-Exo-mediated NRF2 upregulation (Figures 6(a) and 6(b)) and nuclear translocation (Figure 6(c)). In ML385-treated aged PDLSCs, PDLSC-Exos did not reduce SA-*β*-gal activity (Figures 6(d) and 6(e)) or the expression levels of senescence-related proteins (Figures 6(f)–6(h)) and SASP genes (Figure 6(i)). Moreover, ML385 significantly blocked the decrease in oxidative stress mediated by PDLSC-Exos (Figures 6(j)–6(m)). This improvement in the functional phenotype of aged PDLSCs was next examined in relation to NRF2 signaling. After the addition of ML385, the ability of PDLSC-Exos to improve the biological functions of senescent PDLSCs, such as cell proliferation, migration, and differentiation, was significantly inhibited (Supplementary Figure 2). Based on these results, PDLSC-Exos inhibit PDLSC senescence and delay the aging phenotype by promoting NRF2 accumulation and nuclear translocation and lowering ROS levels.

### 3.5. PDLSC-Exos Activate NRF2 by Transferring miR-141-3p to Downregulate KEAP1 Expression

**Figure 7:**
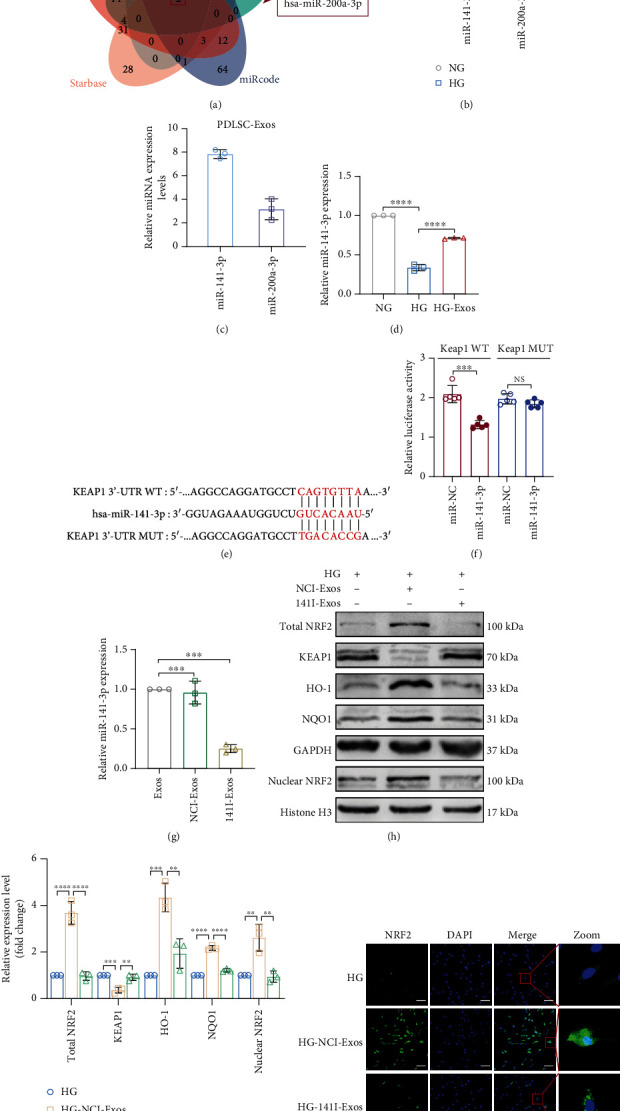
miR-141-3p transferred by PDLSC-Exos activates NRF2 signaling by reducing *KEAP1* expression. (a) Venn diagram showing the miRNAs (hsa-miR-141-3p and hsa-miR-200a-3p) predicted to target the *KEAP1* gene. (b) The expression levels of miRNAs in young and senescent PDLSCs were measured by qRT–PCR. (c) The expression levels of miRNAs in PDLSC-Exos were measured by qRT–PCR. (d) miR-141-3p expression was measured by qRT–PCR analysis. (e, f) Verification of the targeting relationship between miR-141-3p and *KEAP1* using a dual-luciferase reporter gene assay. (g) qRT–PCR analysis was used to determine the expression levels of miR-141-3p in exosomes from treated human PDLSCs. NCI-Exos or 141I-Exos were used to treat high glucose-induced senescent human PDLSCs (HG-NCI-Exos group or HG-141I-Exos group), while untreated aged PDLSCs served as the control (HG group). (h, i) Total NRF2, nuclear NRF2, KEAP1, HO-1, and NQO1 protein levels were measured using WB and quantified using ImageJ software. (j) NRF2 expression levels were quantified, and NRF2 localization was identified by performing IF staining. Scale bar, 100 *μ*m. All data are presented as the means ± SDs (*n* = 3 independent observations). NS indicates not significant, ^∗^*p* < 0.05, ^∗∗^*p* < 0.01, ^∗∗∗^*p* < 0.001, and ^∗∗∗∗^*p* < 0.0001. The corresponding control is indicated.

In subsequent trials, we further investigated the potential mechanism of NRF2 activation. KEAP1 negatively regulates NRF2 activity; therefore, we detected KEAP1 protein expression. KEAP1 expression increased in senescent PDLSCs but decreased following PDLSC-Exo treatment (Figures 5(e) and 5(f)), suggesting that PDLSC-Exos might activate NRF2 signaling by suppressing KEAP1 expression. According to previous reports, exosome-encapsulated miRNAs might be delivered into recipient cells to modify their function by posttranscriptionally controlling the expression of the host gene [40]. By consulting the relevant literature and performing an integrative bioinformatic analysis, we discovered that miR-141-3p and miR-200a-3p are associated with periodontal disease and regulate NRF2 activity by targeting *KEAP1* expression (Figure 7(a)). We measured the expression levels of these two miRNAs in young and senescent PDLSCs and in PDLSC-Exos to further validate the molecular mechanism by which PDLSC-Exos regulate the NRF2 signaling pathway. Compared with those of young PDLSCs (NG group), the expression levels of miR-141-3p and miR-200a-3p in senescent PDLSCs (HG group) were decreased, and the decrease in miR-141-3p levels was more significant (Figure 7(b)). In addition, the expression level of miR-141-3p in PDLSC-Exos was higher than that of miR-200a-3p (Figure 7(c)). Senescent PDLSCs displayed significantly increased expression of miR-141-3p following incubation with PDLSC-Exos (Figure 7(d)), consistent with the downregulation of KEAP1 expression. The results from the dual-luciferase reporter assay revealed that the cells cotransfected with pmiR-*KEAP1*-WT and miR-141-3p mimics showed significantly decreased luciferase reporter gene activity. In cells cotransfected with miR-141-3p mimics and pmiR-*KEAP1*-MUT, no reduction in luciferase activity was observed (Figures 7(e) and 7(f)). These results revealed a clear targeting relationship between miR-141-3p and *KEAP1*.

**Figure 8:**
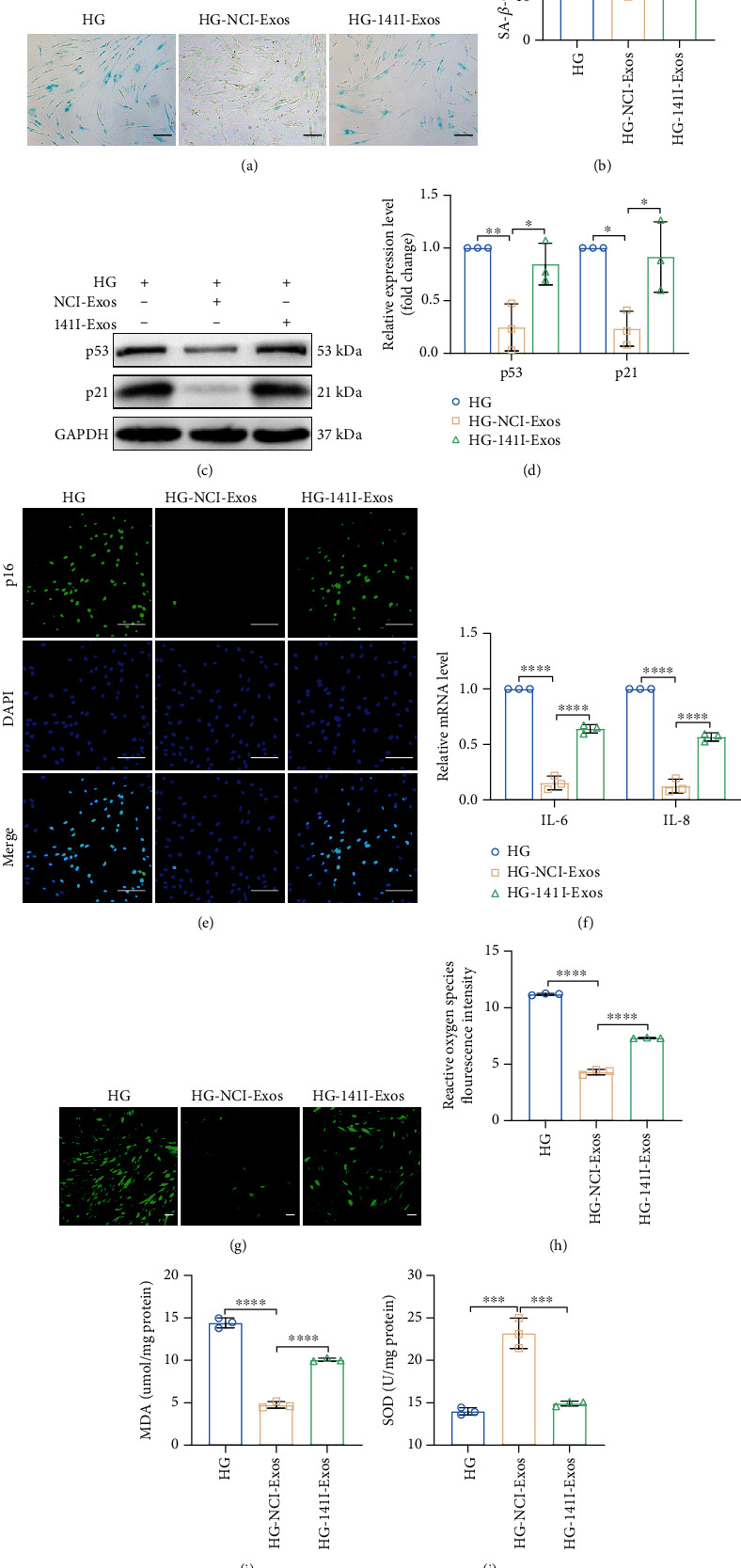
Exosomal miR-141-3p from PDLSCs is essential for preventing PDLSC senescence caused by high-glucose treatment. (a, b) Representative image of SA-*β*-gal staining along with the percentage of SA-*β*-gal-positive cells. Scale bar, 100 *μ*m. (c, d) The expression levels of p53 and p21 were analyzed using WB. (e) Expression of p16 detected using IF staining. Scale bar, 100 *μ*m. (f) Relative levels of the SASP genes *IL-6* and *IL-8*. (g, h) Cellular ROS levels were detected and quantified by DCFH-DA staining. Scale bar, 100 *μ*m. (i, j) MDA levels and SOD activity were assessed to evaluate the level of oxidative stress in cells. All data are presented as the means ± SDs (*n* = 3 independent observations). ^∗^*p* < 0.05, ^∗∗^*p* < 0.01, ^∗∗∗^*p* < 0.001, and ^∗∗∗∗^*p* < 0.0001. The corresponding control is indicated.

**Figure 9:**
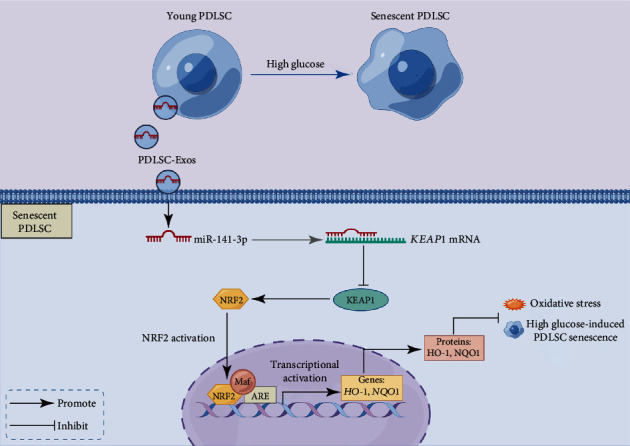
Schematic of the rejuvenating effects of exosomes produced by PDLSCs. High glucose levels accelerated cellular senescence in PDLSCs. PDLSC-Exos protected against high glucose-induced PDLSC senescence by transferring miR-141-3p to senescent PDLSCs to decrease *KEAP1* expression and activate the NRF2 antioxidant pathway. (This image was created using Figdraw).

We then downregulated miR-141-3p expression in PDLSC-Exos (Figure 7(g)) and cocultured them with senescent PDLSCs to investigate the function of exosomal miR-141-3p in improving high glucose-induced PDLSC senescence. Knockdown of miR-141-3p nearly completely reversed the PDLSC-Exo-mediated downregulation of KEAP1 expression and upregulation of NRF2 expression (Figures 7(h)–7(j)). Moreover, the downregulation of miR-141-3p blocked the rejuvenating effects of PDLSC-Exos on the senescent phenotype (SA-*β*-gal activity, senescence-associated protein expression, and SASP level) (Figure 8) and biological functions (proliferation, migration, and multidirectional differentiation) (Supplementary Figure 3) of senescent PDLSCs. These findings indicate that miR-141-3p is one of the major mediators by which PDLSC-Exos activate NRF2 through the suppression of KEAP1 expression to regenerate aged PDLSCs (Figure 9).

## 4. Discussion

Diabetes is a long-term condition characterized by hyperglycemia and is accompanied by multiple comorbidities, such as periodontitis. Studies have suggested that diabetes affects the incidence and severity of periodontitis [2]. In recent decades, the search for effective treatments for diabetic periodontitis has continuously attracted widespread attention. Due to advances in tissue engineering and regenerative medicine, periodontal tissue regeneration utilizing autologous MSCs has emerged as the preferred technique for treating severe periodontal defects [41]. The most promising stem cells for periodontal tissue engineering are PDLSCs [42]. However, some evidence suggests that hyperglycemia may compromise the proliferation and osteogenic capacities of PDLSCs [4]. Alternatively, several studies have identified hyperglycemia as an extracellular stress trigger that induces cellular senescence. Senescent cell accumulation in aging tissues inhibits tissue function and impacts neighboring cells via the SASP [43, 44]. However, to date, the effect of chronic hyperglycemia induced by diabetes on PDLSC senescence has rarely been reported. In this study, we cultured PDLSCs in a 25 mM high-glucose medium to mimic the diabetic microenvironment in periodontal sites. We showed that PDLSCs with sustained high glucose stimulation tended to undergo senescence compared with those cultured in a normal medium, as evidenced by the increases in SA-*β*-gal activity, the expression of senescence-associated proteins (p16, p21, and p53) and SASP (*IL-6* and *IL-8*), and the markedly impaired proliferation, migration, and differentiation. These results suggest that premature senescence is induced in PDLSCs by high glucose levels, which is consistent with other reports showing that high glucose levels induce senescence in other cell types.

Exosomes are endogenous nanovesicles with bilayer membranes. Their biological functions are similar to those of their derived cells but without obvious immunogenicity [45]. Recently, the ability of MSC-Exos to ameliorate senescence has attracted increasing interest. MSC-Exos have been suggested to promote the growth and osteogenic differentiation of senescent bone marrow MSCs [46]. As important participants in the exchange of information between cells, exosomes may be the key substance mediating the role of PDLSCs. In this study, in vitro coculture of PDLSC-Exos and senescent PDLSCs revealed a notable effect on ameliorating the senescent phenotype and biological functions of PDLSCs. The osteogenic differentiation of PDLSCs is crucial for the repair of periodontal tissue defects [42], and we noted a significant decrease in the osteogenic differentiation of high glucose-induced senescent PDLSCs, but PDLSC-Exos reversed this alteration to a certain extent. Based on these results, PDLSC-Exos might protect PDLSCs from high glucose-induced aging and damage.

The free radical theory of aging suggests that the accumulation of physiological damage caused by oxidative stress leads to aging during the normal lifespan of an animal [47]. Therefore, we examined the level of oxidative stress in senescent PDLSCs before and after PDLSC-Exo treatment. High glucose-induced senescent PDLSCs had significantly higher levels of ROS and MDA, despite displaying reduced SOD activity, indicating increased oxidative stress. PDLSC-Exo treatment reversed these changes. Accordingly, one of the main mechanisms by which PDLSC-Exos prevent cellular senescence under high-glucose conditions is probably by reducing oxidative stress. NRF2 is a key transcription factor that regulates antioxidative stress and is crucial for triggering an antioxidant response [25, 48]. Accumulating evidence suggests that NRF2 activity and levels decrease with age, which has been verified in various types of cells, including vascular [49], cardiac [50], epithelial cells [51], and fibroblasts [52]. Selective activation of NRF2 ameliorates or even reverses cellular senescence [53, 54]. Therefore, the NRF2 signaling pathway may be a potential target for reducing cellular senescence. Our results showed that the expression of NRF2 and its corresponding downstream proteins HO-1 and NQO1 was downregulated in senescent PDLSCs, and treatment with PDLSC-Exos restored these changes. Moreover, we observed that PDLSC-Exos promoted the nuclear translocation of NRF2. Further studies revealed that ML385, an inhibitor of NRF2, counterbalanced the improved viability of aged PDLSCs induced by PDLSC-Exo treatment. Therefore, PDLSC-Exos delayed PDLSC senescence in a high-glucose environment via *NRF2* activation, but further studies are needed to determine how PDLSC-Exos upregulate NRF2 expression.

NRF2 activity is regulated by the anchoring protein KEAP1 [55]. In the resting state, NRF2 binds to KEAP1 and is localized within the cytoplasm in its inactivated form, maintaining the low transcriptional activity of NRF2 under physiological conditions. Activation of the NRF2 antioxidant signaling pathway depends on the dissociation of NRF2 from KEAP1 in the cytosol. This process occurs by decreasing KEAP1 expression, which reduces the binding of NRF2 to KEAP1 [23, 56]. Free NRF2 then accumulates to high levels and enters the nucleus as a third messenger, and NRF2 activation induces the transcription of the HO-1 and NQO1 genes encoding antioxidant enzymes, all of which eventually activate the cellular antioxidant process [57]. Several studies have documented a substantial increase in KEAP1 levels in senescent cells, and overexpression of KEAP1 is presumed to alter NRF2 activity in elderly individuals. In contrast, increased NRF2 activity is partially due to a decrease in KEAP1 expression [58]. According to previous reports, activation of the KEAP1-NRF2 signaling pathway enhances cell protection and delays cellular senescence [59]. In the present study, KEAP1 was expressed at high levels in senescent PDLSCs, but its expression level decreased significantly after PDLSC-Exo treatment. However, the details of the mechanism by which PDLSC-Exos target KEAP1, the cytosolic repressor of NRF2, and result in the upregulation of NRF2 target genes are not clear, which became our focus. Exosomes were previously shown to transport and transfer the miRNAs they carry into target cells and participate in intercellular communication [60]. This regulation mainly occurs by inhibiting the translation of mRNAs or mRNA degradation to change gene expression and biological activity in recipient cells [61]. According to numerous studies, certain miRNAs can control NRF2 activity by specifically targeting *KEAP1* expression [62–64]. Here, we examined the expression of the screened miRNAs in PDLSC-Exos and chose miR-141-3p, which had a comparatively greater expression, for additional investigation. After the downregulation of miR-141-3p expression in PDLSC-Exos, PDLSC-Exos were unable to increase NRF2 activity while decreasing the expression of KEAP1. The recovery effect produced by PDLSC-Exos in senescent PDLSCs was also largely but not completely eliminated by downregulating exosomal miR-141-3p. These findings indicate that miR-141-3p is an essential mediator of PDLSC-Exo-mediated senescent PDLSC regeneration and that it activates NRF2 by suppressing *KEAP1* expression. Importantly, although miR-141-3p was downregulated in PDLSC-Exos, the efficacy of PDLSC-Exos in activating NRF2 and decreasing PDLSC senescence remained, suggesting that additional antisenescence mechanisms may be involved, which requires further investigation.

## 5. Conclusions

In this study, we showed that high levels of glucose accelerate premature senescence in PDLSCs and that PDLSC-Exos promote the regeneration of senescent PDLSCs. PDLSC-Exos may exert antiaging effects by transferring miR-141-3p to downregulate *KEAP1* expression and activate the NRF2 antioxidant pathway. Our research suggests that PDLSC-Exos may behave similarly to their parental PDLSCs and exert significant effects on cellular senescence by delivering their encapsulated bioactive chemicals to target cells.

## Data Availability

The data used to support the findings of this study are available from the corresponding author upon request.

## Supplementary Materials

Supplementary Materials Abbreviations. Supplementary Table 1: antibodies used in this study. Supplementary Table 2: mRNA primers used for qRT-PCR. Supplementary Table 3: miRNA primers used for qRT-PCR. Supplementary Figure 1: the function of high glucose-induced senescent PDLSCs is impaired. Supplementary Figure 2: PDLSC-Exos enhance the biological functions of senescent PDLSCs in an NRF2-dependent manner. Supplementary Figure 3: downregulation of miR-141-3p inhibits the ability of PDLSC-Exos to restore the biological function of senescent PDLSCs.
