# Supplementary material for: Exosomal miR-141-3p from PDLSCs Alleviates High Glucose-Induced Senescence of PDLSCs by Activating the KEAP1-NRF2 Signaling Pathway
Source: Stem Cells Int. 2023 May 26;2023:7136819. doi: 10.1155/2023/7136819 (PMC10238146; doi:10.1155/2023/7136819)
Supplement: Supplementary Materials — Abbreviations. Supplementary Table 1: antibodies used in this study. Supplementary Table 2: mRNA primers used for qRT-PCR. Supplementary Table 3: miRNA primers used for qRT-PCR. Supplementary Figure 1: the function of high glucose-induced senescent PDLSCs is impaired. Supplementary Figure 2: PDLSC-Exos enhance the biological functions of senescent PDLSCs in an NRF2-dependent manner. Supplementary Figure 3: downregulation of miR-141-3p inhibits the ability of PDLSC-Exos to restore the biological function of senescent PDLSCs. [file 7136819.f1.zip › Supplementary Table 1 (1).docx]

**Supplementary Table 1: Antibodies used in this study.**

| Antibodies | Source | Dilution |
| --- | --- | --- |
| p53 | Cell Signaling Technology Cat# 2527 | 1:1000 (WB) |
| p21 | Cell Signaling Technology Cat# 2947 | 1:1000 (WB) |
| p16 | Affinity Biosciences Cat# AF5484, RRID: AB_2837964 | 1:200 (IF) |
| NRF2 | Affinity Biosciences Cat# AF0639, RRID: AB_2833793 | 1:1000 (WB)  1:200 (IF) |
| KEAP1 | Affinity Biosciences Cat# AF5266, RRID: AB_2837752 | 1:1000 (WB) |
| HO-1 | Affinity Biosciences Cat# AF5393, RRID: AB_2837878 | 1:1000 (WB) |
| NQO1 | Affinity Biosciences Cat# DF6437, RRID: AB_2838400 | 1:1000 (WB) |
| GAPDH | Affinity Biosciences Cat# AF7021, RRID: AB_2839421 | 1:1000 (WB) |
| Histone H3 | Affinity Biosciences Cat# AF0863, RRID: AB_2810277 | 1:1000 (WB) |
| CD9 | Abcam Cat# ab263019 | 1:1000 (WB) |
| CD63 | Abclonal Cat# A19023 | 1:1000 (WB) |
| CD81 | Abcam Cat# ab109201 | 1:1000 (WB) |
| TSG101 | Abcam Cat# ab125011 | 1:1000 (WB) |
| Calnexin | Abcam Cat# ab133615 | 1:1000 (WB) |
