# Supplementary material for: Exosomal miR-141-3p from PDLSCs Alleviates High Glucose-Induced Senescence of PDLSCs by Activating the KEAP1-NRF2 Signaling Pathway
Source: Stem Cells Int. 2023 May 26;2023:7136819. doi: 10.1155/2023/7136819 (PMC10238146; doi:10.1155/2023/7136819)
Supplement: Supplementary Materials — Abbreviations. Supplementary Table 1: antibodies used in this study. Supplementary Table 2: mRNA primers used for qRT-PCR. Supplementary Table 3: miRNA primers used for qRT-PCR. Supplementary Figure 1: the function of high glucose-induced senescent PDLSCs is impaired. Supplementary Figure 2: PDLSC-Exos enhance the biological functions of senescent PDLSCs in an NRF2-dependent manner. Supplementary Figure 3: downregulation of miR-141-3p inhibits the ability of PDLSC-Exos to restore the biological function of senescent PDLSCs. [file 7136819.f1.zip › Supplementary Table 3 (1).docx]

**Supplementary Table 3: MiRNA primers used for qRT-PCR.**

| miRNA | Sequences |
| --- | --- |
| miR-141-3p | Forward (5′–3′): GCGCTAACACTGTCTGGTAAAGATGG |
|  | Reverse (5′–3′): AACGCTTCACGAATTTGCGT |
| miR-200a-3p | Forward (5′–3′): GCGTAACACTGTCTGGTAACGATGT |
|  | Reverse (5′–3′): AACGCTTCACGAATTTGCGT |
| U6 | Forward (5′–3′): CTCGCTTCGGCAGCACA |
|  | Reverse (5′–3′): AACGCTTCACGAATTTGCGT |
