# Supplementary material for: Exosomal miR-141-3p from PDLSCs Alleviates High Glucose-Induced Senescence of PDLSCs by Activating the KEAP1-NRF2 Signaling Pathway
Source: Stem Cells Int. 2023 May 26;2023:7136819. doi: 10.1155/2023/7136819 (PMC10238146; doi:10.1155/2023/7136819)
Supplement: Supplementary Materials — Abbreviations. Supplementary Table 1: antibodies used in this study. Supplementary Table 2: mRNA primers used for qRT-PCR. Supplementary Table 3: miRNA primers used for qRT-PCR. Supplementary Figure 1: the function of high glucose-induced senescent PDLSCs is impaired. Supplementary Figure 2: PDLSC-Exos enhance the biological functions of senescent PDLSCs in an NRF2-dependent manner. Supplementary Figure 3: downregulation of miR-141-3p inhibits the ability of PDLSC-Exos to restore the biological function of senescent PDLSCs. [file 7136819.f1.zip › The Revised Supplementary Material (1).docx]

**Abbreviations**

ALP: Alkaline phosphatase; BCA: bicinchoninic acid; CCK8: Cell Counting Kit-8; DAPI: 4′,6-Diamidino-2-phenylindole; HO-1: Heme oxygenase 1; IF: Immunofluorescence; IL-6: Interleukin 6; IL-8: Interleukin 8; KEAP1: Kelch-like ECH associated protein 1; MDA: Malondialdehyde; MiRNAs: MicroRNAs; MiR-141-3p: MicroRNA-141-3p; MiR-200a-3p: MicroRNA-200a-3p; MSCs: Mesenchymal stem cells; MSC-Exos: Mesenchymal stem cell-derived exosomes; MUT, Mutant; NQO1: NADPH quinone oxidoreductase 1; NRF2: Nuclear factor erythroid 2-related factor 2; NTA: Nanoparticle tracking analysis; PBS: Phosphate-buffered saline; PDLSCs: Periodontal ligament stem cells; PDLSC-Exos: Periodontal ligament stem cell-derived exosomes; PFA: Paraformaldehyde; SA-β-gal: Senescence-associated β-galactosidase; SASP: Senescence-associated secretory phenotype; SOD: Superoxide dismutase; SD: Standard deviation; TEM: Transmission electron microscopy; UTR, Untranslated region; WB: Western blotting; WT: Wild-type.

**Supplementary Table 1: Antibodies used in this study.**

| Antibodies | Source | Dilution |
| --- | --- | --- |
| p53 | Cell Signaling Technology Cat# 2527 | 1:1000 (WB) |
| p21 | Cell Signaling Technology Cat# 2947 | 1:1000 (WB) |
| p16 | Affinity Biosciences Cat# AF5484, RRID: AB_2837964 | 1:200 (IF) |
| NRF2 | Affinity Biosciences Cat# AF0639, RRID: AB_2833793 | 1:1000 (WB)  1:200 (IF) |
| KEAP1 | Affinity Biosciences Cat# AF5266, RRID: AB_2837752 | 1:1000 (WB) |
| HO-1 | Affinity Biosciences Cat# AF5393, RRID: AB_2837878 | 1:1000 (WB) |
| NQO1 | Affinity Biosciences Cat# DF6437, RRID: AB_2838400 | 1:1000 (WB) |
| GAPDH | Affinity Biosciences Cat# AF7021, RRID: AB_2839421 | 1:1000 (WB) |
| Histone H3 | Affinity Biosciences Cat# AF0863, RRID: AB_2810277 | 1:1000 (WB) |
| CD9 | Abcam Cat# ab263019 | 1:1000 (WB) |
| CD63 | Abclonal Cat# A19023 | 1:1000 (WB) |
| CD81 | Abcam Cat# ab109201 | 1:1000 (WB) |
| TSG101 | Abcam Cat# ab125011 | 1:1000 (WB) |
| Calnexin | Abcam Cat# ab133615 | 1:1000 (WB) |

**Supplementary Table 2: MRNA primers used for qRT-PCR.**

| mRNA | Sequences |
| --- | --- |
| IL-6 | Forward (5′–3′): GTTGCCTTCTTGGGACTGATG |
|  | Reverse (5′–3′): TTGGGAGTGGTATCCTCTGTGA |
| IL-8 | Forward (5′–3′): TGGGTGAAGGCTACTGTTGG |
|  | Reverse (5′–3′): AGAGGCTTTTCATGCTCAACAC |
| GAPDH | Forward (5′–3′): CGACCACTTTGTCAAGCTCA |
|  | Reverse (5′–3′): CCCTGTTGCTGTAGCCAAAT |

**Supplementary Table 3: MiRNA primers used for qRT-PCR.**

| miRNA | Sequences |
| --- | --- |
| miR-141-3p | Forward (5′–3′): GCGCTAACACTGTCTGGTAAAGATGG |
|  | Reverse (5′–3′): AACGCTTCACGAATTTGCGT |
| miR-200a-3p | Forward (5′–3′): GCGTAACACTGTCTGGTAACGATGT |
|  | Reverse (5′–3′): AACGCTTCACGAATTTGCGT |
| U6 | Forward (5′–3′): CTCGCTTCGGCAGCACA |
|  | Reverse (5′–3′): AACGCTTCACGAATTTGCGT |

**Supplementary Figure 1:** **The function of high glucose-induced senescent PDLSCs is impaired. A-B** The ability of PDLSCs to proliferate was assessed using CCK-8 assays and plate colony formation tests. Scale bar, 100 μm. Cell migration was examined by performing wound healing **C-D** and Transwell assays **E-F**. Scale bar, 100 μm. **G-H** PDLSCs were employed to assess osteogenesis after 7 days of induction using ALP staining and an activity assay. Scale bar, 100 μm. **I-J** Calcium deposition was evaluated using alizarin red staining and measurements. Scale bar, 100 μm. **K-L** Oil red O staining and subsequent quantification revealed the ability of the cells in the three groups to differentiate into adipocytes. Scale bar, 100 μm. All data are presented as the means ± SDs (n = 3 independent observations). *p < 0.05, ***p < 0.001, and ****p < 0.0001. The corresponding control is indicated.

**Supplementary Figure 2:** **PDLSC-Exos enhance the biological functions of senescent PDLSCs in an NRF2-dependent manner.** **A** Cell proliferation was assayed using CCK-8 assays. **B** Cell clone formation was evaluated by performing colony formation assays. Scale bar, 100 μm. Cell migration was assessed using wound healing assays **C-D** and Transwell experiments **E-F**. Scale bar, 100 μm. **G-H** ALP staining and activity assays were used to evaluate PDLSC osteogenesis after 7 days of induction. Scale bar, 100 μm. **I-J** With alizarin red staining and subsequent quantification, calcium deposition was evaluated after osteogenic stimulation for 21 days. Scale bar, 100 μm. **K-L** Oil red O staining was used to detect cell adipogenic differentiation. Scale bar, 100 μm. All data are presented as the means ± SDs (n = 3 independent observations). NS represents not significant, *p < 0.05, **p < 0.01, ***p < 0.001, and ****p < 0.0001. The corresponding control is indicated.

**Supplementary Figure 3:** **Downregulation of miR-141-3p inhibits the ability of PDLSC-Exos to restore the biological function of senescent PDLSCs.** High glucose-induced senescent PDLSCs were divided into three groups: the HG group (PBS treatment), the HG-NCI-Exos group (50 μg/mL NCI-Exo treatment, exosomes isolated from PDLSCs transfected with the miR-141-3p inhibitor negative control), and the HG-141I-Exos group (50 μg/mL 141I-Exo treatment, exosomes isolated from PDLSCs transfected with the miR-141-3p inhibitor). **A-B** Cell proliferation was assessed using CCK-8 and colony formation assays. Scale bar, 100 μm. Cell migration was examined by performing wound healing **C-D** and Transwell assays **E-F.** Scale bar, 100 μm. **G-H** ALP staining and activity assays. Scale bar, 100 μm. **I-J** Alizarin red staining was implemented to identify and measure calcium deposition. Scale bar, 100 μm. **K** Oil red O staining. Scale bar, 100 μm. **L** Quantification of oil red O staining. All data are presented as the means ± SDs (n = 3 independent observations). *p < 0.05, **p < 0.01, ***p < 0.001, and ****p < 0.0001. The corresponding control is indicated.
